# Preparation of nano-selenium from chestnut polysaccharide and characterization of its antioxidant activity

**DOI:** 10.3389/fnut.2022.1054601

**Published:** 2023-01-18

**Authors:** Shanshan Wang, Hao Wu, Xiaoshuang Zhang, Shihong Luo, Shuang Zhou, Haiyan Fan, Chunmao Lv

**Affiliations:** ^1^College of Bioscience and Biotechnology, Shenyang Agricultural University, Shenyang, China; ^2^Food Science College, Shenyang Agricultural University, Shenyang, China

**Keywords:** chestnut polysaccharide, ultrasonic processing, polysaccharide purifier, nano-selenium polysaccharide, antioxidant activity

## Abstract

Chestnut is widely cultivated and has high nutritional value due to its richness in polysaccharides. In order to improve the antioxidant activity of chestnut polysaccharide, chestnut polysaccharide (CP) was extracted by ultrasonic-assisted water extraction and alcohol precipitation and purified by cellulose DEAE-52 exchange and Sephadex G-100 chromatography in this study. CP isolates were characterized by I_2_-KI reaction, three-strand helical structure analysis, infrared spectrum analysis, and nuclear magnetic resonance detection. The results showed that CP is a pyrylan sugar with triple helical structure and connected by α-glycosidic bonds, with sugar residues 1,4-α-D-Glcp, 1,6-α-D-Galp, 1,5-α-L-Araf, 1,4-α-L-Rhap, and 1,4-β-D-Glcp in the CP backbone. After purification, the branching structure, rod, and spherical structure were significantly increased, with reduced lamellar structure. The *in vitro* scavenging rates of CP at 10 mg·mL^−1^ against DPPH, hydroxyl radicals, and ABTS were 88.95, 41.38, and 48.16%, respectively. The DPPH free radical scavenging rate of purified polysaccharide fraction CP-1a was slightly enhanced, and the other rates showed a small decrease. Selenized chestnut polysaccharide (CP-Se) was prepared using nano-selenium method. The selenization method was optimized and stable Se-CP was obtained. When the concentration was 5 mg·mL^−1^, Se-CP had significantly higher scavenging abilities 89.81 ± 2.33, 58.50 ± 1.60, and 40.66 ± 1.91% for DPPH, hydroxyl radical, and ABTS radicals, respectively, than those of CP. The results of this study provide insight into the effects purification and selenization of chestnut polysaccharide on antioxidant activity, and also provide a theoretical basis for the development of chestnut polysaccharide for use in functional foods or health products.

## Introduction

Chinese chestnut (*Castanea mollissima* BL.) fruit is rich in polysaccharides, flavonoids, polyphenols, and other bioactive substances, enabling its use as medicine and food. China leads the world in both the planting area and yield of Chinese chestnut (1). Chestnut is primarily used as a food and a polysaccharide (2). Polysaccharides are natural chain-like polymer formed by glycosidic bonds with a stable structure that is maintained during processing. Polysaccharides are used as natural and non-toxic food additives, and allow emulsification, enhance water holding capacity of food, and improve food stability (3). Natural polysaccharides may have antioxidant, hypoglycemic, anti-aging, anti-tumor, antibacterial, and other biological activities (4). Chestnut kernels contain more than 15% polysaccharides. Recent work has described the extraction and purification of chestnut polysaccharides (5, 6), however, different extraction methods and varieties of chestnut result in different efficiencies of polysaccharide extraction (7). Studies of chestnut polysaccharide have mainly focused on optimization of the extraction, separation, and purification steps from the shell and coat of chestnut (8, 9), but despite the high abundance of polysaccharides in chestnut seeds, the best ways to extract polysaccharides from the seeds have not been determined. Further, although molecular modification can improve the activity and stability of polysaccharides, there have been few studies of the activity, structure, and chemical modification of chestnut seed polysaccharides.

Selenium is a trace element required for human survival and an important component of glutathione peroxidase, which promotes the oxidation and decomposition of glutathione and reduction of harmful peroxides (10). As the particle size of selenium decreases, the antioxidant and tumor inhibitory activities are improved (11, 12). Selenium-modified polysaccharides (Se-polysaccharides) retain the activity of original inorganic selenium and the physiological function of polysaccharides, with greater stability than the unmodified polysaccharide material. Free radicals produced during cell metabolism cause degenerative changes during the aging process. A variety of active substances in plants, such as polyphenols and polysaccharides, can play antioxidant roles (13). Se-polysaccharides can improve the activity of glutathione peroxidase for improved ability to remove free radicals and reduce damage from reactive oxygen species (14, 15). Se-polysaccharides can also protect B cells of pancreatic islets and improve insulin sensitivity (16). Overall, selenium polysaccharides have impressive antioxidant, anticancer, and hypoglycemic properties, suggesting a broad application prospect in the fields of medicine and health.

The goals of this work were to optimize the extraction of chestnut polysaccharides, test the effect of selenium modification of the polysaccharides, and investigate the antioxidant capacity and structure of the modified polysaccharides. The results showed that nano-selenium modification significantly and stably improved the antioxidant activity of chestnut polysaccharides, providing the scientific basis and theoretical support for further applications of this material.

## 2. Materials and methods

### 2.1. Materials and reagents

Chinese chestnut (Dandong Dafeng), Phenol, sulfuric acid, 1,1-diphenyl-2-picrylhydrazyl (DPPH), 2,2'-azinobis-(3-ethyl-benzothiazolin-6-sulfonic acid) diammonium salt (ABTS), Congo red, and Iodine were obtained from Sinopharm Group, Dialysis tubing (3,000 kDa), Vitamin C, Potassium iodide, DEAE Cellulose DE-52, and Glucan gel G-100 were obtained from Solarbio. All reagents were obtained as analytically pure (AR).

### 2.2. Extraction of crude polysaccharides from Chinese chestnut

Dried powder of whole chestnut seeds was weighed and extracted three times with different material: liquid ratio, ultrasonic time, extraction time, and extraction temperature. Samples were centrifugated for 15 min at 9,500 r/min to get the supernatant, and then five times the volume of 95% ethanol was added into supernatant at 4°C and incubated for 24 h before centrifuging for 10 min at 9,500 r/min. The resulting precipitate was redissolved and added Sevag (Chloroform: n-butanol=4:1) to remove protein, dialyzed in ultrapure water for 3 days, and then dried to obtain dried Chestnut crude polysaccharide.

### 2.3. Optimization of ultrasonic extraction of chestnut crude polysaccharide

#### 2.3.1. Single factor experimental design

Ultrasonic extraction of chestnut polysaccharide was performed. The main factors varied in the extraction of chestnut polysaccharide were solid-liquid ratio, ultrasonic time, extraction time, and extraction temperature. To explore the influence of these factors on the yield of chestnut polysaccharide, the extraction rate of chestnut polysaccharide was used as an index and the above factors were tested by single factor analysis. The factors and levels of the single factor experiment are shown in Supplementary Table 1.

#### 2.3.2. Response surface experimental design

According to the single factor test results and Central Composite Design (CCD) principle, Design Expert 12.0 software was used for the orthogonal experimental design. Extraction temperature (A), solid-liquid ratio (B), ultrasonic time (C), and extraction time (D) were selected as investigation factors (Supplementary Table 2). A mathematical model between response value and influencing factors was established to screen parameters and optimize the extraction process of chestnut polysaccharide.

#### 2.3.3. Validation experimentation

Chestnut dry powder was prepared according to the optimal conditions of the single factor and response surface experiments, and the chestnut polysaccharide content of the samples was determined by phenol-sulfuric acid method (17). Three parallel tests were conducted and the average value was calculated.

### 2.4. Chestnut crude polysaccharide purification

Gradient elution of polysaccharides was carried out by cellulose DEAE-52 column chromatography with NaCl concentrations of 0, 0.1, 0.3, and 0.5 mol·L^−1^, using a flow rate of 1.0 mL/min with 4 mL samples collected. The absorbance value at 490 nm was determined for each sample by sulfuric acid-phenol method, and the chromatographic elution curve was drawn. Eluents were combined and the resulting isolated chestnut polysaccharide was desalted and freeze-dried.

Sephadex G-100 column chromatography was used, with 0.1 mol·L^−1^ NaCl for elution at an elution rate of 0.25 mL·min^−1^, and 4 mL samples were collected. The absorbance value at 490 nm was determined by sulfuric acid-phenol method and the elution curve was drawn. According to the elution curve, the eluents were combined, and the resulting isolated chestnut polysaccharide was dialyzed, desalted, and freeze-dried.

### 2.5. Structural characterization

#### 2.5.1. Determination of molecular weight of chestnut polysaccharide

The molecular weights of chestnut polysaccharides CP and CP-1a were determined by high performance gel permeation chromatography (HPGPC) as described.

#### 2.5.2. Determination of monosaccharide composition of chestnut polysaccharide

The monosaccharide composition of chestnut polysaccharide was determined by ion chromatograph according to the method of Li et al. (18). Chromatographic analysis conditions: column, Dionex CarbopacTMPA20 (3^*^150); mobile phase, A: H_2_O, B: 250 mmol·L^−1^ NaOH, C: 50 mmol·L^−1^ NaOH and 500 mmol·L^−1^ NaOAC; flow rate: 0.3 mL·min^−1^; injection volume, 5 μL; column temperature: 30°C; detector, electrochemical detector.

#### 2.5.3. NMR measurement

DRX NMR instrument was used to detect the polysaccharides according to the method of Liu et al. (19). ^1^H NMR, ^13^C NMR, HSQC, HMBC, ^1^H-^1^H COSY, and NOESY were analyzed on the 600 MHz NMR instrument at room temperature. Mestrenova-11.0.2 software was used to analyze and process the data.

#### 2.5.4. I_2_-KI reaction

The I_2_-KI method was used to determine the relative amount of side chains of polysaccharides based on the method of Wang et al. (20) with some modifications. Solutions of CP, CP-1, and CP-1a were prepared at 2.0 mg·mL^−1^, 200 μL samples of chestnut polysaccharide solutions were added to the wells of a microplate, and 80 μL iodine reagent was added to each well. After 10 min at room temperature, the absorbance in the range of 300–700 nm was measured with a microplate reader.

#### 2.5.5. Determination of triple helix structure

The chestnut polysaccharide conformation was determined by Congo red method according to the methods of Hu et al. (21) and Song et al. (22). First, 150 μL of 2.0 mg·mL^−1^ polysaccharide solution sample was transferred into a 2.0 mL centrifuge tube, and then an equal volume of 100 μmol·L^−1^ Congo red solution was added and mixed. Next, two volumes (600 μL) of NaOH solution with concentrations of 0, 0.05, 0.1, 0.15, 0.2, 0.25, 0.3, 0.35, 0.4, 0.45, and 0.5 mol·L^−1^ were added and incubated for 15 min at room temperature. Scanning was performed with a microplate reader (wavelength range: 400–600 nm).

#### 2.5.6. Infrared spectroscopy

The infrared detection of polysaccharides was carried out using the KBr tablet pressing method, with treatment of the polysaccharide material according to the methods of Wu et al. (23). Briefly, air was used as a reference, and the blank slices were compressed with potassium bromide for interval scanning of 400–4,000 cm^−1^.

#### 2.5.7. Determination of microstructure

Scanning electron microscopy (SEM) was used to observe the surface morphology of the crude and purified chestnut polysaccharides. According to the method of Huang et al. (24), appropriate amounts of freeze-dried polysaccharide samples (CP, CP-1, and CP-1a) were glued to the conductive adhesive of the test bed, and gold spraying was carried out under vacuum condition. The electron gun acceleration voltage was 15 kV, and the observation was carried out under 200, 500, 1,000, 2,000, and 5,000 multiples.

### 2.6. Assay of antioxidant activity

The scavenging activities of un-modified polysaccharide and nano-Se polysaccharide were determined for DPPH, hydroxyl, and ABTS radicals (25–28). The experiment included a blank group, sample experimental group, and sample control group. The polysaccharide concentrations tested were 1.0, 2.0, 3.0, 4.0, 5.0, 6.0, 7.0, 8.0, 9.0, and 10.0 mg·mL^−1^, and the Se-polysaccharide concentrations were 1.0, 2.0, 3.0, 4.0, and 5.0 mg·mL^−1^. The experiment was repeated three times with ascorbic acid (Vc) as the positive control and average values were calculated.


$$\begin{array}{l} Clearance\;rate=\frac{A_{0}-\left(A_{1}-A_{2}\right)}{A_{0}} \end{array}$$

Where, A_0_ is absorbance of anhydrous ethanol as the blank group absorbance, A_1_ is the absorbance of the polysaccharide test sample, A_2_ is the absorbance of a control sample with anhydrous ethanol in place of DPPH, ultrapure water instead of hydrogen peroxide, or absolute ethanol instead of ABTS.

### 2.7. Selenization of chestnut crude polysaccharide

Selenization was performed according to the Wang et al. (29) method. Briefly, chestnut polysaccharide (50 mg·mL^−1^) was added to a beaker and mixed with sodium selenite solution (100 mg·mL^−1^). The same volume of ascorbic acid solution (305.5 mg·mL^−1^) was added, and then the volume of distilled water was adjusted to 5 mL. The reaction solution was mixed on a magnetic stirrer for 4 h at a certain temperature. The reaction was then centrifuged at 4,000 rpm/min for 20 min. After centrifugation, the samples were dialyzed with a 3,000 kDa dialysis bag in ultrapure water for 48 h (water was changed every 12 h), and the dialyzed nano-selenium polysaccharide solution was then lyophilized to obtain nano-selenium polysaccharides (Se-CP) (30, 31).

### 2.8. Stability determination of selenized chestnut polysaccharide

Nano-selenium polysaccharides have the basic properties of a colloid. For a colloidal solution, the stability is closely related to the size of the colloidal particle, that is, the smaller the particle size, the higher the stability. Therefore, the absorbance of Se-chestnut polysaccharides can be measured by the dual-wavelength method to assess a size change of nano-selenium particles (32–35).

### 2.9. Optimization of selenized chestnut polysaccharide

#### 2.9.1. Single factor experiment

The four factors of ultrasonic time, chestnut polysaccharide concentration, sodium selenite concentration, and selenization temperature were tested for effects on yield of Se-CP. The DPPH clearance rate was used as the index, and the above factors were tested by single factor experiment, as shown in Supplementary Table 3.

#### 2.9.2. Response surface test

According to the single factor test results and the Central Composite Design (CCD) Design principle, orthogonal experiment design was carried out using the Design Expert 12.0 software. The ultrasonic time, chestnut polysaccharide concentration, sodium selenite concentration, and selenization temperature were selected as the investigation factors, and the mathematical model between the response value and the influencing factors was established. The experimental design factors and levels are shown in Supplementary Table 4.

#### 2.9.3. Validation experimentation

The single factor (stability) condition and the single factor (clearance rate) condition were used. Validation experimentation was conducted with the optimal conditions obtained from the response surface test. Three parallel tests were conducted and the average value was calculated.

## 3. Results and discussion

### 3.1. Optimization of crude polysaccharide extraction process

#### 3.1.1. Analysis of single factor test results

**Figure 1:**
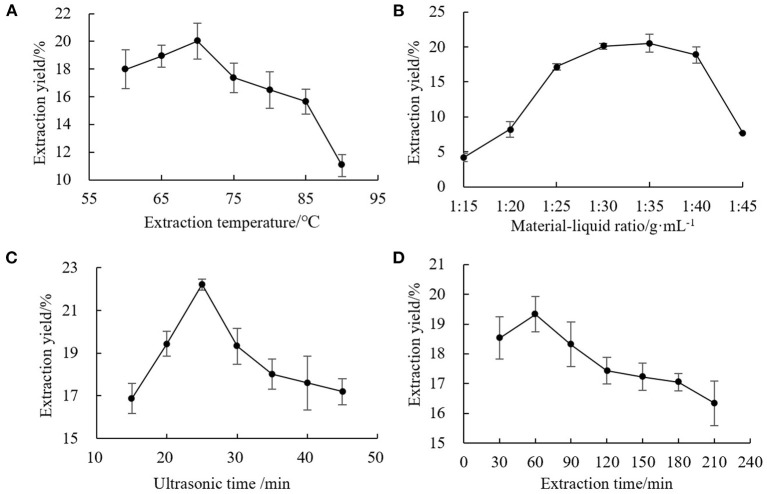
Effects of single factors on extraction yield of chestnut polysaccharide **(A)** extraction temperature, **(B)** solid-liquid ratio, **(C)** ultrasonic time, and **(D)** extraction time.

For the single factor experiments, one factor was varied while the others were kept constant. The experimental results are shown in Figure 1. The single factor optimization conditions were as follows: extraction temperature, 70°C; solid-liquid ratio, 1:35 g·mL^−1^; ultrasonic time, 25 min; extraction time, 60 min. These parameters were used in subsequent optimization tests. With the improvement of the level of influencing factors, the extraction solvent might dissolve other substances at the same time. The structure of the chestnut polysaccharide chain could be destroyed or decomposed by high temperature and longtime ultrasonic wave and thermal effect, thus reducing the extraction effect.

#### 3.1.2. Response surface test analysis

To more carefully analyze the optimal conditions for chestnut polysaccharide extraction, response surface methodology was used to explore the relationships between the four explanatory variables and the extraction rate as the response variable. In the response surface test, extraction temperature (A), solid-liquid ratio (B), ultrasonic time (C), and extraction time (D) were tested at five levels with the experimental design established using CCD of Design-Expert 12.0 software. The response surface analysis scheme and results are shown in Supplementary Table 5. Multiple regression fitting was conducted on the experimental data in Supplementary Table 6 to obtain a regression equation with the extraction rate of chestnut polysaccharide as the response value:


$$\begin{array}{l} Extraction\;yield\;of\;chestnut\;polysaccharide\;=18.53+0.5121A\\ +1.31B+0.7807C+0.4952D+0.6907AB-0.8886AC\\ +0.8313AD-0.2725BC-1.60BD+0.1539CD-0.0912A^{2}\\ -0.4035B2+0.2012C^{2}-0.4013D^{2} \end{array}$$

The response surface analysis scheme and experimental results showed that the regression model was extremely significant (*P* < 0.01), and the misfit term was not significant (*P* > 0.05). The coefficient of determination of the regression model was *R*^2^ = 0.9778, and the adjustment coefficient was $R_{\text{Adj}}^{2}$ = 0.9571, indicated a good fit of the regression model with the experimental data, suggested this model could be used for theoretical prediction of the extraction rate of chestnut polysaccharide. According to the significance, all four factors had extremely significant effects on the extraction of chestnut polysaccharide. The influence degree was in the order of solid-liquid ratio > ultrasonic time > extraction temperature > extraction time. The interaction terms AD, BC, and BD were extremely significant (*P* < 0.01) (<0.0001 in Supplementary Table 5). The quadratic terms B^2^ and D^2^ (*P* < 0.01) and C^2^ (*P* < 0.05) had significant effects on the extraction rate of chestnut polysaccharide.

**Figure 2:**
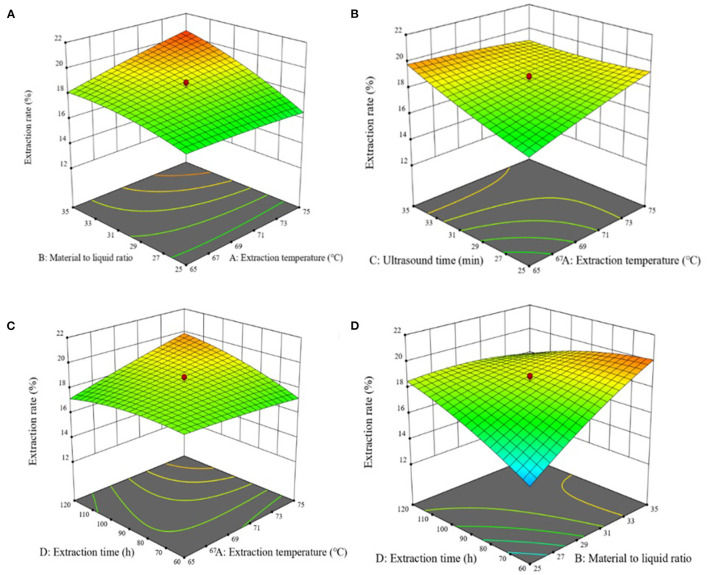
Effects of response on extraction yield of chestnut polysaccharides **(A)** ultrasonic time and sodium selenite concentration **(B)** polysaccharide concentration and selenization temperature **(C)** sodium selenite concentration and selenization temperature **(D)** sodium selenite concentration and selenization temperature.

The response surface of chestnut polysaccharide extraction process optimization is shown in Figure 2. AB, AD, AC, and BD showed highly significant interaction (*P* < 0.001) among the independent variables, with highly significant effects on the extraction of chestnut polysaccharide. Expert Design 12 software was used to analyze the experimental results, and the optimal extraction conditions of chestnut polysaccharide were obtained: extraction temperature, 75°C; solid-liquid ratio, 1:35 g·mL^−1^; ultrasonic time, 25 min; extraction time, 74.278 min; with a predicted extraction rate of chestnut polysaccharide of 21.246%. For practical operation, the extraction conditions were simplified as extraction temperature, 75°C; solid-liquid ratio, 1:35 g·mL^−1^; ultrasonic time, 25 min; and extraction time, 74 min.

#### 3.1.3. Validation experimentation of the conditions for chestnut polysaccharide

**Figure 3:**
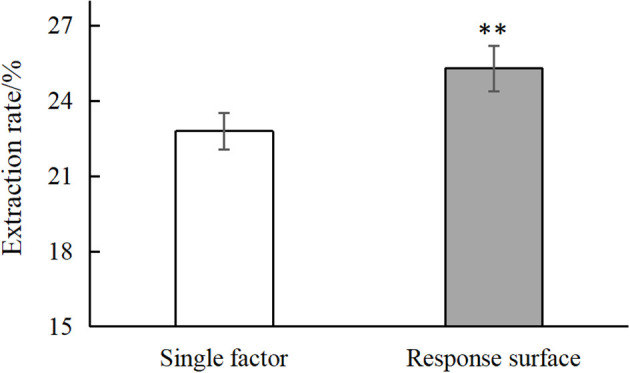
Comparison of single factor and response surface. “**” means very significant difference (*P* < 0.01).

The optimal conditions as determined by the single factor experiments and the response surface test were next validated, as shown in Supplementary Table 7. As shown in Figure 3, the optimal conditions as determined by single factor test gave an average chestnut polysaccharide extraction yield of 22.794% and the conditions determined by the response surface methodology gave an average extraction rate of 25.296%, significantly higher than the yield for the single factor optimization condition and better than the predicted value of Design Expert 12.0. Overall, the results demonstrate the successful optimization of the extraction process.

### 3.2. Purification of chestnut crude polysaccharide

#### 3.2.1. Cellulose DEAE-52 chromatography of crude chestnut polysaccharide

The results of cellulose DE-52 column chromatography are shown in Supplementary Figure 1. The chestnut polysaccharide was eluted in ultrapure water and NaCl solutions of different concentrations (0.1, 0.3, and 0.5 mol·L^−1^) and four main polysaccharides components were obtained, named CP, CP-1, CP-2, and CP-3. The polysaccharides of each component were dialyzed, concentrated, and freeze-dried. CP-1 was the main component, while the content of CP-2 and CP-3 were very low.

#### 3.2.2. Sephadex G-100 chromatography of crude chestnut polysaccharide

Since this fraction exhibited the best antioxidant activity and content, the CP-1 material was further subjected to Sephadex G-100 column chromatography and the results are shown in Supplementary Figure 2. CP-1 showed a single component absorption peak, but there was tailing phenomenon. After dialysis, concentration and freeze-drying, a white flocculent was obtained, which was named CP-1a.

### 3.3. *In vitro* antioxidant activity analysis of chestnut polysaccharide

**Figure 4:**
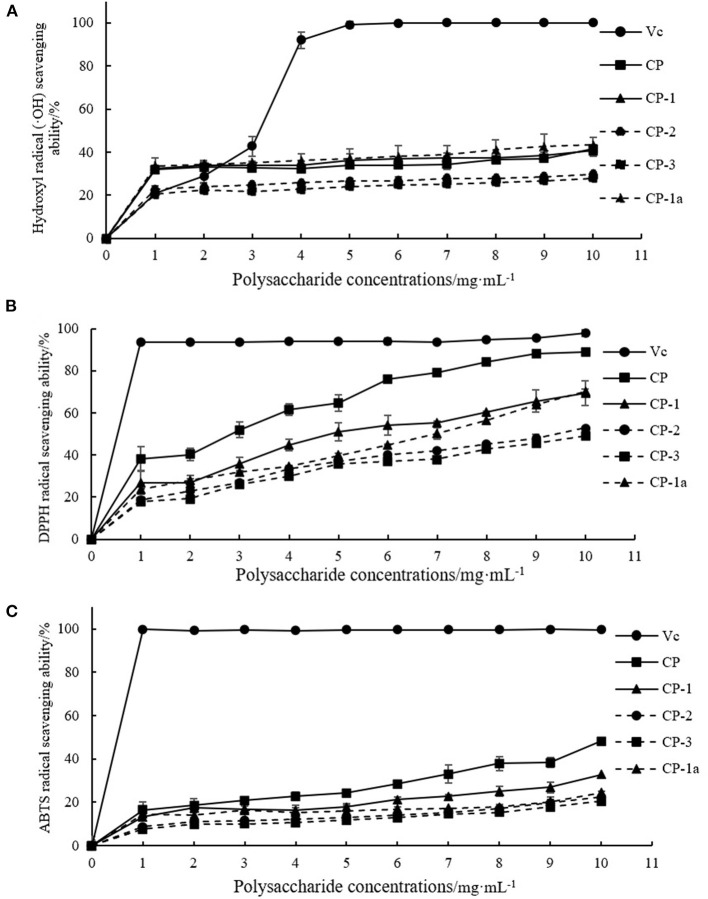
Antioxidant capacity of purified chestnut polysaccharide **(A)** DPPH free radical scavenging capacity **(B)** hydroxyl radical scavenging capacity **(C)** ABTS free radical scavenging capacity.

Within the selected concentration range, the five (CP, CP-1, CP-2, CP-3, CP-1a) polysaccharides exhibited dose-dependent scavenging of DPPH, hydroxyl, and ABTS radicals as shown in Figure 4. When the concentration of CP was 4 mg·mL^−1^, the scavenging ability of DPPH radical reached 60.50 ± 2.78%, which was higher than the maximum value of lycium barbarum polysaccharide (50.12%) (36). The scavenging ability of hydroxyl radical reached 32.27 ± 1.07%, which was higher than the maximum value of sea cucumber polysaccharide (23.45%) (37). When the concentration of CP was 10 mg·mL^−1^, the scavenging ability of CP on ABTS radical reached 48.16 ± 2.26%, which was higher than the maximum value of sorghum polysaccharide (about 40.00%) (38). At this concentration, the scavenging ability of CP on DPPH radical reached 88.95 ± 1.22%, and the scavenging ability on hydroxyl radical reached 41.38 ± 6.03%.

The CP-2 and CP-3 fractions exhibited poor activity. After purification, CP-1a had improved scavenging of hydroxyl radicals, but weakened scavenging of DPPH and ABTS free radicals. This may be caused by the removal of some other substances with antioxidant activity (such as polyphenols) or the destruction of some of the polysaccharide structure during purification (39–42). The ultrasonic treatment might oxidize the hydroxyl group, ultimately reducing activity (43–45).

### 3.4. Structure characterization of chestnut polysaccharide

#### 3.4.1. Composition analysis of crude and purified chestnut polysaccharide

Compositional analysis of CP revealed a polysaccharide composition of rhenose (0.9): arabinose (4.1): (0.2): galactose (5.7): glucose (84.6): galacturonic acid (4.6), with a molecular weight of 1.6 × 105 Da. CP-1a polysaccharide is composed of arabinose (0.7): galactose (2.4): glucose (96.9) with a molecular weight of 1.3 × 105 Da. The results are shown in Supplementary Figures 3–5.

Ultraviolet analysis showed no absorption peaks at 260 and 400–700 nm, indicating that the sample does not contain nucleic acids or pigment. However, a small absorption peak was observed at 280 nm, indicating the presence of a small amount of protein, as shown in Supplementary Figure 6.

#### 3.4.2. NMR analysis

**Figure 5:**
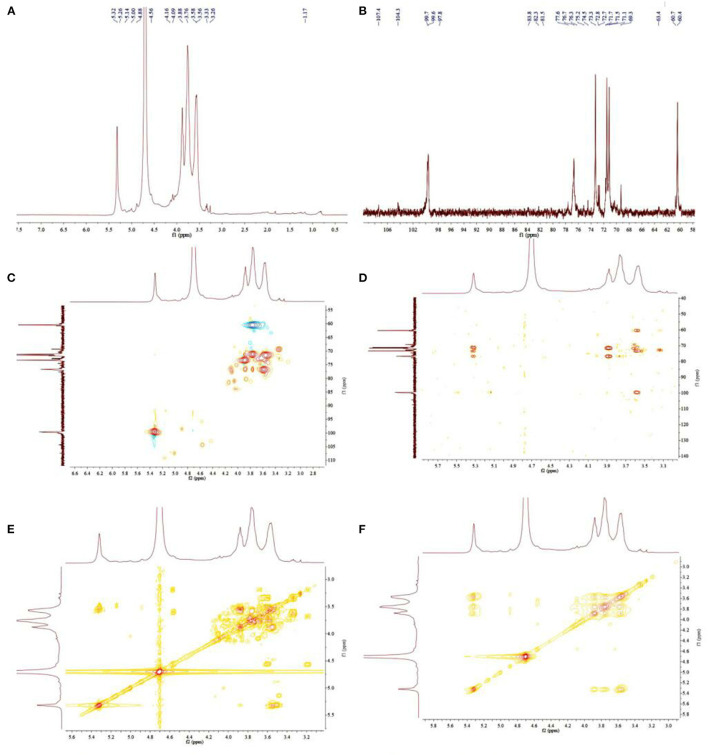
^1^H NMR **(A)**,^13^C NMR **(B)**, HSQC **(C)**, HMBC **(D)**, ^1^H-^1^H COSY **(E)**, and NOESY **(F)** spectra of CP.

^1^H NMR, ^13^C NMR, HSQC, HMBC, ^1^H-^1^H COSY, and NOESY were used to characterize the monosaccharide residues and heteromorphic carbon configurations in chestnut polysaccharides. The sugar residues and monosaccharide linkages in chemical structural features of CP were demonstrated from the analyses of 1D and 2D NMR spectroscopy. The prominent six carbon signals in 13C NMR at δC 99.6, 71.5, 73.3, 76.7, 71.1, and 60.4 suggested a glucopyranosyl group in CP through the data comparison with the literature (46, 47) (Table 1). According to HSQC spectrum analyses, these carbon resonances C-1 to C-6 are completely corresponding to δH 5.32, 3.56, 3.88, 3.57, and 3.76, which was attributed to H-1, H-2, H-3, H-4, H-5, and H-6, respectively (Figure 5). The small coupling constant value was showed in anomeric proton signal at δH 5.32, indicating the typical substituent of alpha-D glucopyranosyl moieties. A downfield shift of value + 6.1 ppm for C-4 (δC 76.7) was characteristic of → 4)-α-D-Glcp-(1 → (48, 49). In the heteronuclear multiple bond coherence (HMBC) spectrum of CP (Figure 5), the 1H-13C correlations from the anomeric protons H-1 (δH 5.32) to C-2 and C-3, and H2-6 (δH 3.76) to C-4 and C-5, demonstrated the prominent glycosidic linkages as → 4)-α-D-Glcp-(1 →. Furthermore, through anomeric terminal based signals C-1/H-1 at values of 99.7/5.28, 107.4/5.01, 97.4/5.31, and 104.3/4.56, and further detailed analyses of HSQC and HMBC spectra, the sugar residue moieties of → 6)-α-D-Galp-(1 →, → 5)-α-L-Araf-(1 →, → 4)-α-L-Rhap-(1 →, and → 4)-β-D-Glcp-(1 → were also documented in the backbone of CP (Table 1 and Figure 5). Thus, sugar residues 1,4-α-D-Glcp, 1,6-α-D-Galp, 1,5-α-L-Araf, 1,4-α-L-Rhap, and 1,4-β-D-Glcp were existed in the backbone of CP.

**Table 1:** ^1^H and ^13^C NMR chemical shifts (ppm) for the polysaccharide of CP in D_2_O.

**Sugar residues**	**C-1/H-1**	**C-2/H-2**	**C-3/H-3**	**C-4/H-4**	**C-5/H-5**	**C-6/H-6**
→ 4)-α-D-Glc*p*-(1→	99.6/5.32	71.5/3.56	73.3/3.88	76.7/3.57	71.1/3.75	60.4/3.76
→ 6)-α-D-Gal*p*-(1→	99.7/5.28	71.4/3.88	72.7/3.61	71.7/3.59	72.8/3.65	69.3/3.35
→ 5)-α-L-Ara*f*-(1→	107.4/5.01	81.5/4.06	76.7/4.07	83.8/4.02	63.4/3.47	-
→ 4)-α-L-Rha*p*-(1→	97.4/5.31	76.3/3.86	69.3/4.01	77.6/4.09	70.4/3.83	16.5/1.17
→ 4)-β-D-Glc*p*-(1→	104.3/4.56	73.2/3.55	75.2/4.00	78.5/3.57	74.5/3.65	60.7/3.67

#### 3.4.3. I_2_-KI reaction of chestnut polysaccharide

**Figure 6:**
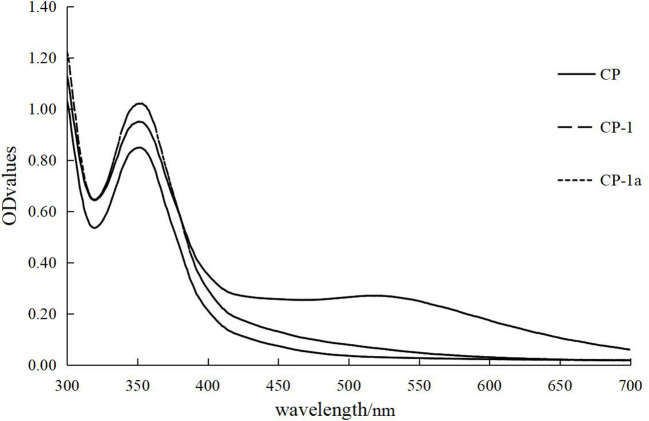
I_2_-KI reaction of CP, CP-1, and CP-1a.

As shown in Figure 6, CP has a maximum absorption peak at 565 nm, and CP-1 and CP-1a lack absorption peaks at this position. Compared with CP, the purified polysaccharide has a relatively long sugar chain and more side chains, which may reflect the destruction of the original polysaccharides structure and the reduction of oligosaccharides in the crude sample during the purification process. Some of the original short chain structure may be destroyed, causing the relative amount of long fragments to increase.

#### 3.4.4. Analysis of triple helix structure

**Figure 7:**
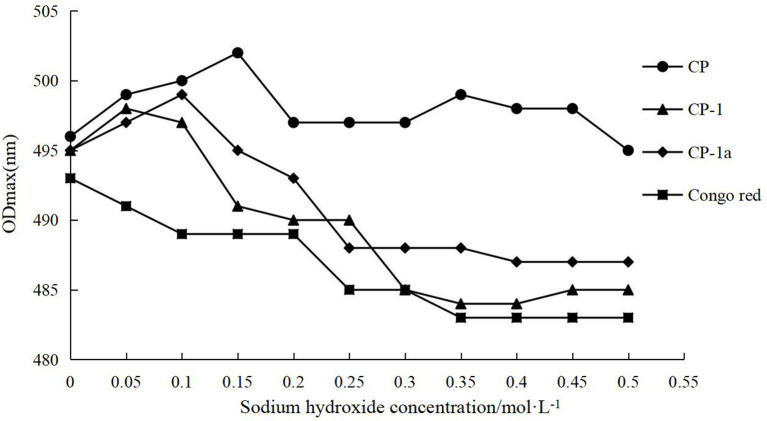
Triple helix conformation analysis of CP, CP-1, CP-1a, and Congo red.

The variation of the maximum absorption wavelength of polysaccharides and Congo red complex were determined and the results are shown in Figure 7. The crude and purified polysaccharide samples all bound Congo red, indicating that the samples maintain regular helical conformations. The red shift distance of CP was significantly larger than that of CP-1 and CP-1a, indicating the helical degree of CP was more complex. This may reflect the partial destruction of the original helical structure after purification, leading to a reduction of red shift distance (50).

#### 3.4.5. Infrared spectrum analysis

**Figure 8:**
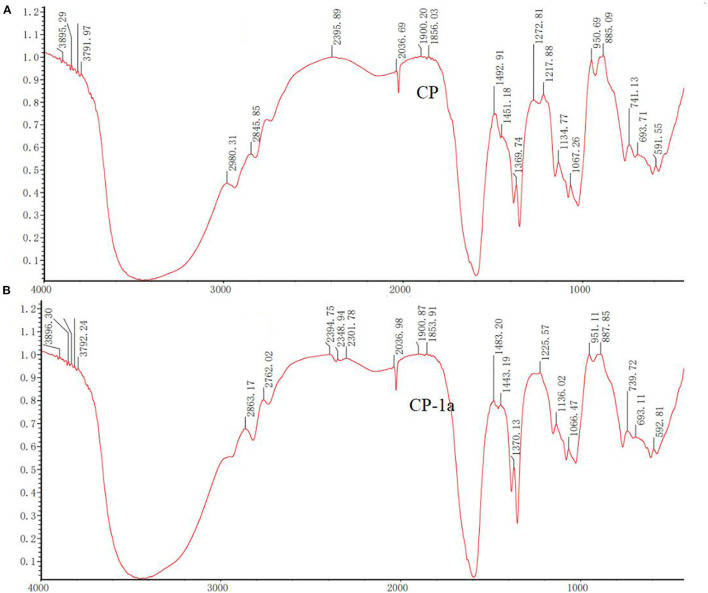
Infrared spectra of CP **(A)** and CP-1a **(B)**.

The chemical bonds and functional groups of chestnut polysaccharides CP and CP-1a were determined by FT-IR technology (51, 52). Figure 8 shows that the absorption peak between 950 and 1,160 cm^−1^ is generated by the tensile vibration of pyranose ring (53–57). The characteristic absorption peak near 870 cm^−1^ showed linkage of chestnut polysaccharides by α-glycosidic bonds (58). Compared with CP, the infrared spectrum of CP-1a changed, with several weak signal peaks stretched by aliphatic C-H bonds split at 2,400–2,300 cm^−1^ and increased peak area at 1,400–1,000 cm^−1^. These results show that the purified polysaccharide material has different internal structure compared to the crude polysaccharide sample.

#### 3.4.6. SEM analysis

**Figure 9:**
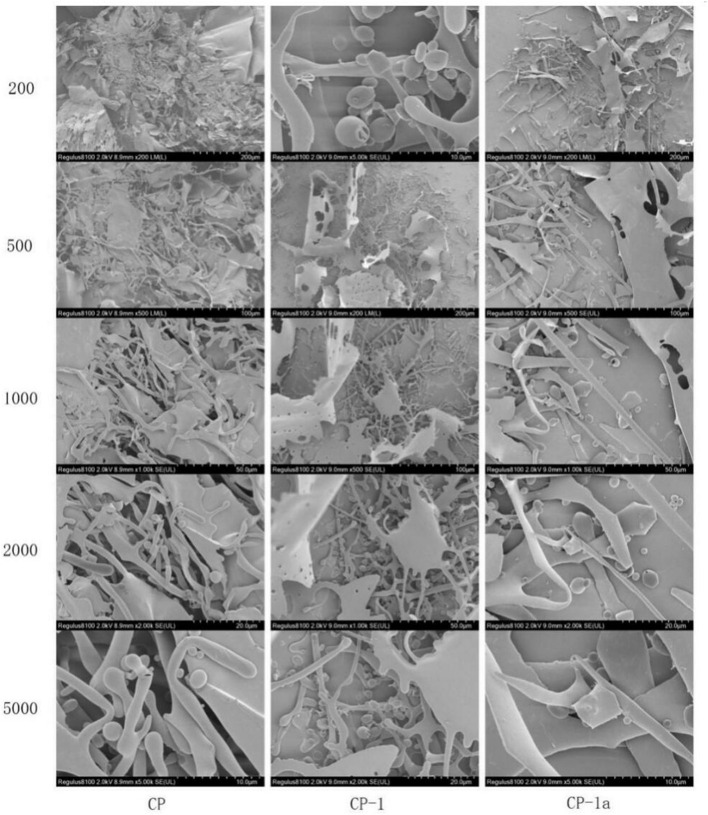
Scanning electron microscopy of CP, CP-1, and CP-1a.

SEM analysis was performed for CP, CP-1 and CP-1a (CP-2 and CP-3 were not included due to low antioxidant activity) and Figure 9 shows the 200 to 5,000 SEM magnification images of the three polysaccharide samples. At 1,000, 2,000, and 5,000 magnification, the surface structure of polysaccharides was observed as smooth, with increased rod-like structure, and the uneven distribution of smooth spherical particles.

The surface microstructure of CP-1 did not appear significantly different from that of CP-1a, but was significantly different from that of CP. This is mainly manifested in the reduced fragment layer structure of the purified polysaccharide sample, and is due to the damage caused by ultrasonic treatment (59, 60). The rod-like and spherical structures were significantly increased by ultrasonic treatment, due to the turbulent shear and instantaneous high pressure, which caused cell wall rupture.

### 3.5. Stability of selenized chestnut polysaccharide

**Figure 10:**
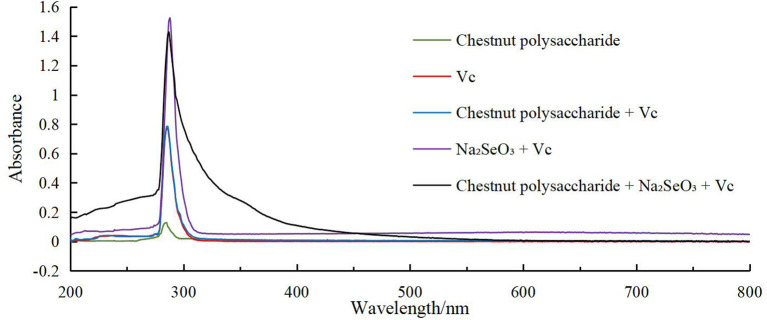
Full wavelength scanning result chart.

To determine the stability of nano-Se polysaccharide, the absorbance values of the experimental group (chestnut polysaccharide+Na_2_SeO_3_+Vc) and the control group (chestnut polysaccharide, Vc, chestnut polysaccharide+Vc, and Na_2_SeO_3_+Vc) in the range of 200–800 nm were scanned by dual-wavelength colorimetric method, as shown in Figure 10. When the ratio of absorbance values at two wavelengths (A1/A2) was unchanged, the particle size of nano-selenium did not change. The larger the ratio, the smaller the particle size of nano-selenium, and the more stable the morphology. Therefore, the A410/A490 value was selected as the standard to evaluate the stability of Se-CP.

### 3.6. Optimization of nano-selenium chestnut polysaccharide technology

#### 3.6.1. Univariate experimental analysis

**Figure 11:**
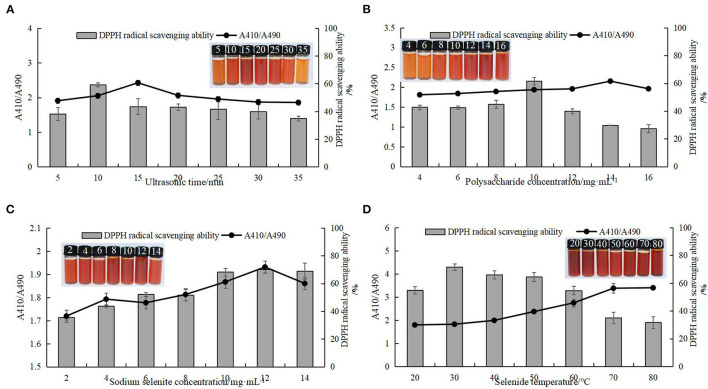
Stability of selenated chestnut polysaccharide and DPPH free radical scavenging ability **(A)** ultrasonic time **(B)** polysaccharide concentration **(C)** sodium selenite concentration **(D)** selenation temperature.

Single factor testing was performed to determine the effects on the preparation of selenized chestnut polysaccharide, as measured by stability of A410/A490 and DPPH scavenging ability. As shown in Figure 11, with the continuous increase of the four influencing factors, the corresponding stability and DPPH free radical scavenging ability of Se-CP showed a trend of first increasing and then decreasing. As the stability of Se-CP increased, its color gradually changed from orange to brown-red. Too long ultrasonic time, high temperature, and high concentration of polysaccharides can break the major chemical bonds in Se-CP, thus affecting its stability and leading to the decrease of DPPH clearance rate (61–64).

#### 3.6.2. Analysis of response surface data and determination of the optimum extraction process of Se-chestnut polysaccharide

According to the single factor test results, A410/A490 and the scavenging rate of DPPH free radicals were selected as the response values for a CCD experiment with four factors and five levels of ultrasonic time (A), chestnut polysaccharide concentration (B), sodium selenite concentration (C) and selenization temperature (D). The response surface analysis scheme and results are shown in Supplementary Table 8. Multiple regression fitting was performed on the experimental data using Design-Expert 12.0 software, and the regression equation with DPPH radical clearance rate as response value was obtained as follows:


$$\begin{array}{l} A410/A490=1.99+0.0083A+0.0185B+0.051C+0.1807D\\ +0.0048AB-0.0276AC-0.0031AD+0.0018BC-0.0292BD\\ +0.0377CD-0.0003A^{2}-0.0035B^{2}+0.0207C^{2}+0.0431D^{2} \end{array}$$

$$\begin{array}{l} DPPH\;clearance\;rate\;=53.21+1.59A-6.1B+7C+\\ 3.34D-0.1503AB+0.2306AC+0.2573AD+0.0112BC\\ +0.8085BD+4.03CD+0.1175A^{2}+1.59B^{2}+0.1145C^{2}+3.89D^{2} \end{array}$$

As shown in Supplementary Table 9, analysis of the variance of A410/A490 regression equation shows that the regression model was extremely significant (*P* < 0.0001), and the misfit term was not significant (*P* > 0.05). The coefficient of determination of the regression model was *R*^2^ = 0.9846, and the adjustment coefficient was $R_{\text{Adj}}^{2}$ = 0.9703, indicating that the regression model fitted well with the experimental data and could be used for theoretical prediction of the stability of Se-chestnut polysaccharide. The significance test of regression equation coefficient showed that the influence degree of each factor on the extraction yield of chestnut polysaccharide was in the order of selenization temperature > sodium selenite concentration > chestnut polysaccharide concentration > ultrasonic time. The sodium selenite concentration, chestnut polysaccharide concentration, and ultrasonic time had extremely significant effects on the stability of Se-chestnut polysaccharide (*P* < 0.01). The interaction terms AC, BD, and CD were extremely significant (*P* < 0.01). The quadratic terms C^2^ and D^2^ had significant effects on the stability of Se-chestnut polysaccharide (*P* < 0.01).

**Figure 12:**
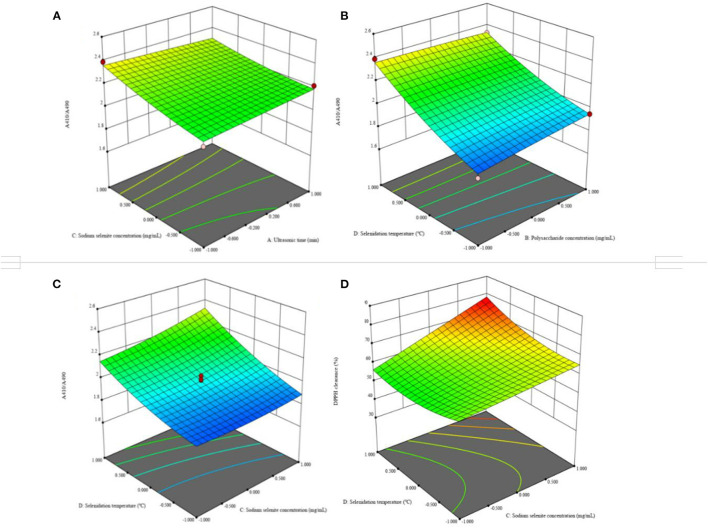
Effects of response on extraction yield of selenized chestnut polysaccharides **(A)** ultrasonic time and sodium selenite concentration **(B)** polysaccharide concentration and selenite temperature **(C)** sodium selenite concentration and selenite temperature **(D)** sodium selenite concentration and selenite temperature.

Supplementary Table 10 shows the results of variance analysis of regression equation of DPPH clearance rate. The regression model was extremely significant (*P* < 0.0001), and the misfit term was not significant (*P* > 0.05). The coefficient of determination of the regression model was *R*^2^ = 0.7927, and the adjustment coefficient was $R_{\text{Adj}}^{2}$ = 0.5993, indicating that the regression model fitted well with the test, and could be used to predict the DPPH scavenging ability of Se-chestnut polysaccharide. The significance test of regression equation coefficient shows that the influence degree of each factor on DPPH scavenging ability is in the order of sodium selenite concentration > chestnut polysaccharide concentration > selenization temperature > ultrasonic time. Figure 12 lists the four items, AB, AD, BC, and BD, with highly significant interaction (*P* < 0.001) among the independent variables. The response surface analysis revealed the optimal extraction conditions of chestnut polysaccharide as 10 min, ultrasonic time; chestnut polysaccharide concentration of 10 mg·mL^−1^; sodium selenite concentration of 12 mg·mL^−1^; and selenide temperature of 50°C.

#### 3.6.3. Validation experimentation

**Figure 13:**
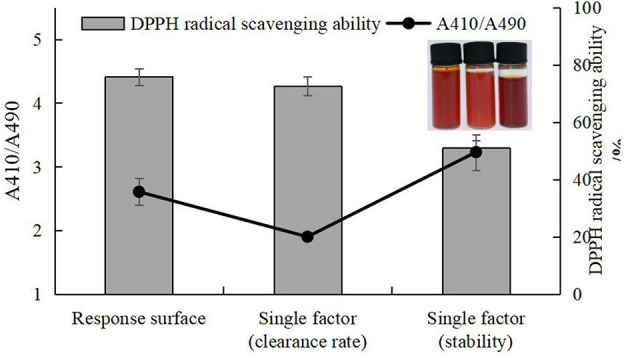
Comparison of selenization conditions of response surface, single factor (clearance rate), and single factor (stability).

The experiment to validate the selenization method was carried out according to the specifications listed in Supplementary Table 11. As shown in Figure 13, there was no significant difference in DPPH clearance rate between the response surface and single factor results. Although there was a significant difference between the response surface, single factor (clearance rate), and single factor (stability) results, the scheme single factor (stability) was excluded due to the low DPPH clearance. Comparison of the response surface and single factor (clearance rate) experiments revealed no significant differences, but the DPPH radical scavenging ability of Se-chestnut polysaccharides prepared according to the response surface scheme was slightly better than that prepared using the single factor optimization conditions and better than the predicted value of Design Expert 12.0. In terms of stability, there were significant differences in the response surface, single factor (clearance), and single factor (stability) results. The response surface solution stability of the prepared Se-chestnut polysaccharide was better than that of the material prepared according to the single factor optimization condition, illustrating these conditions are feasible in practice.

### 3.7. Antioxidant activity analysis of chestnut nano-selenium polysaccharide

**Figure 14:**
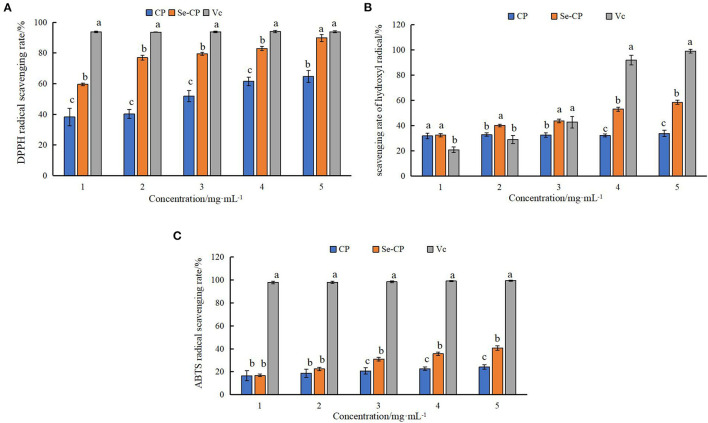
Antioxidant activity of selenated polysaccharides **(A)** DPPH radical scavenging activity **(B)** hydroxyl radical scavenging activity **(C)** ABTS radical scavenging activity.

The analysis of the antioxidant activities of the polysaccharides materials is shown in Figure 14 CP and Se-CP exhibited dose-dependent removal activities of DPPH, hydroxyl, and ABTS radicals. The scavenging activities of Se-CP were similar to CP for hydroxyl and ABTS radicals at lower concentration, but significantly better than CP at higher concentration. Overall, the scavenging activity of Se-CP on DPPH radical was significantly higher than that of CP in the selected concentration range, and at concentration of 1–2 mg·mL^−1^, the scavenging activity of Se-CP on hydroxyl radical was significantly higher than that of Vc (*P* < 0.05). The main chemical bonds of selenated polysaccharides are C-O-Se and Se=O. The results suggest that selenation changed the structure of chestnut polysaccharide to improve its stability and improve its antioxidant ability (65, 66).

## 4. Conclusion

Chestnut polysaccharide (CP) was extracted and purified by ultrasonic assisted water extraction and alcohol precipitation, with an extraction rate of 25.296%. The structural characterization of the purified chestnut polysaccharide showed obvious changes, which might be caused by binding of the large lamellar structure to the cellulose column during the purification process. The *in vitro* scavenging rates of CP at 10 mg·mL^−1^ against DPPH, hydroxyl radicals, and ABTS were 88.95, 41.38, and 48.16%, respectively. The DPPH free radical scavenging rate of purified polysaccharide fraction CP-1a was slightly enhanced, and the other rates showed a small decrease. Se-CP had scavenging abilities of 89.81 ± 2.33, 58.50 ± 1.60, and 40.66 ± 1.91% for DPPH, hydroxyl radicals, and ABTS radicals, respectively, increases of 38.83, 72.73, and 68.19%, respectively, relative to un-modified CP. In conclusion, nano-selenium modification of chestnut polysaccharide can improve antioxidant activity, and there is potential use of this material in the field of food therapy for treatment of insulin resistance. The findings of excellent scavenging suggest a direction for further research on the mechanism of hypoglycemic activity.

## Data availability statement

The original contributions presented in the study are included in the article/Supplementary material, further inquiries can be directed to the corresponding authors.

## Author contributions

SW: conceptualization, methodology, validation, data curation, writing—original draft preparation, visualization, and funding acquisition. HW: methodology, writing—review and editing, software, and validation. XZ and SZ: validation and software. SL: formal analysis and validation. HF: resources, writing—review and editing, and funding acquisition. CL: writing—review and editing, funding acquisition, and supervision. All authors contributed to the article and approved the submitted version.
