# Supplementary material for: Preparation of nano-selenium from chestnut polysaccharide and characterization of its antioxidant activity
Source: Front Nutr. 2023 Jan 18;9:1054601. doi: 10.3389/fnut.2022.1054601 (PMC9889657; doi:10.3389/fnut.2022.1054601)
Supplement: Supplementary file 1 [file Data_Sheet_1.docx]

**Supplementary Table 1.** Factors and levels of single factor test design

| Level | Extraction temperature  /℃ | Solid-liquid ratio  /(g·mL^-1^) | Ultrasound Time  /min | Extraction time  /min |
| --- | --- | --- | --- | --- |
| 1 | 60 | 1:15 | 15 | 30 |
| 2 | 65 | 1:20 | 20 | 60 |
| 3 | 70 | 1:25 | 25 | 90 |
| 4 | 75 | 1:30 | 30 | 120 |
| 5 | 80 | 1:35 | 35 | 150 |
| 6 | 85 | 1:40 | 40 | 180 |
| 7 | 90 | 1:45 | 45 | 210 |

**Supplementary Table 2.** Central Composite Design test with factors and levels

| Factor | Level | | | | |
| --- | --- | --- | --- | --- | --- |
|  | -2 | -1 | 0 | 1 | 2 |
| Extraction temperature(A)/℃ | 60 | 65 | 70 | 75 | 80 |
| Solid-liquid ratio(B)/(g·mL^-1^) | 1:20 | 1:25 | 1:30 | 1:35 | 1:40 |
| Ultrasound Time(C)/min | 20 | 25 | 30 | 35 | 40 |
| Extraction time(D)/min | 30 | 60 | 90 | 120 | 150 |

**Supplementary Table 3.** Factors and levels of single factor experiment design

| level | Ultrasonic Time  /min | Polysaccharide concentration  /mg·mL^-1^ | Sodium selenite concentration  /mg·mL^-1^ | Selenization temperature  /℃ |
| --- | --- | --- | --- | --- |
| 1 | 2 | 4 | 2 | 20 |
| 2 | 10 | 6 | 4 | 30 |
| 3 | 15 | 8 | 6 | 40 |
| 4 | 20 | 10 | 8 | 50 |
| 5 | 25 | 12 | 10 | 60 |
| 6 | 30 | 14 | 12 | 70 |
| 7 | 35 | 16 | 14 | 80 |

**Supplementary Table 4.** Central Composite Design test with factors and levels

| Factor | Level | | | | |
| --- | --- | --- | --- | --- | --- |
|  | -2 | -1 | 0 | 1 | 2 |
| Ultrasound Time(A)/min | 5 | 10 | 15 | 20 | 25 |
| Polysaccharide concentration(B)/(mg·mL^-1^) | 8 | 10 | 12 | 14 | 16 |
| Sodium selenite concentration(C)/(mg·mL^-1^) | 6 | 8 | 10 | 12 | 14 |
| Selenide temperature(D)/℃ | 20 | 30 | 40 | 50 | 60 |

**Supplementary Table 5.** Response surface analysis scheme and test results

| test number | A | B | C | D | Extraction ratio/% |
| --- | --- | --- | --- | --- | --- |
| 1 | 0.000 | 0.000 | 0.000 | 0.000 | 18.8795 |
| 2 | 2.000 | 0.000 | 0.000 | 0.000 | 18.9980 |
| 3 | -2.000 | 0.000 | 0.000 | 0.000 | 17.6201 |
| 4 | 1.000 | 1.000 | 1.000 | -1.000 | 20.4173 |
| 5 | 1.000 | -1.000 | 1.000 | 1.000 | 19.8346 |
| 6 | 1.000 | 1.000 | -1.000 | -1.000 | 20.9628 |
| 7 | -1.000 | 1.000 | -1.000 | 1.000 | 13.9529 |
| 8 | 1.000 | 1.000 | 1.000 | 1.000 | 19.7509 |
| 9 | 1.000 | -1.000 | -1.000 | -1.000 | 13.1201 |
| 10 | 0.000 | 0.000 | 0.000 | 2.000 | 17.7538 |
| 11 | 0.000 | 0.000 | 2.000 | 0.000 | 20.7162 |
| 12 | 0.000 | 0.000 | 0.000 | -2.000 | 16.3829 |
| 13 | -1.000 | 1.000 | 1.000 | -1.000 | 20.7562 |
| 14 | 0.000 | 0.000 | 0.000 | 0.000 | 18.3249 |
| 15 | -1.000 | -1.000 | 1.000 | 1.000 | 19.6533 |
| 16 | 0.000 | 2.000 | 0.000 | 0.000 | 19.2502 |
| 17 | 0.000 | 0.000 | -2.000 | 0.000 | 18.2406 |
| 18 | 1.000 | -1.000 | -1.000 | 1.000 | 18.7166 |
| 19 | 1.000 | -1.000 | 1.000 | -1.000 | 13.3445 |
| 20 | -1.000 | 1.000 | -1.000 | -1.000 | 18.7128 |
| 21 | 0.000 | 0.000 | 0.000 | 0.000 | 18.9600 |
| 22 | -1.000 | -1.000 | -1.000 | 1.000 | 15.8620 |
| 23 | -1.000 | 1.000 | 1.000 | 1.000 | 18.1804 |
| 24 | -1.000 | -1.000 | -1.000 | -1.000 | 13.1600 |
| 25 | 0.000 | 0.000 | 0.000 | 0.000 | 18.4688 |
| 26 | 1.000 | 1.000 | -1.000 | 1.000 | 20.7638 |
| 27 | 0.000 | 0.000 | 0.000 | 0.000 | 18.1493 |
| 28 | 0.000 | 0.000 | 0.000 | 0.000 | 18.4162 |
| 29 | 0.000 | -2.000 | 0.000 | 0.000 | 14.8692 |
| 30 | -1.000 | -1.000 | 1.000 | -1.000 | 17.0991 |

**Supplementary Table 6.** Response surface analysis scheme and experiment results

| Soruce of variation | Quadratic sum | Degree of freedom | Mean square | F value | Pro>F | Significance |
| --- | --- | --- | --- | --- | --- | --- |
| Model | 152.12 | 14 | 10.87 | 47.26 | < 0.0001 | Significant |
| *A* | 6.29 | 1 | 6.29 | 27.37 | 0.0001 | ** |
| *B* | 41.26 | 1 | 41.26 | 179.47 | < 0.0001 | ** |
| *C* | 14.63 | 1 | 14.63 | 63.62 | < 0.0001 | ** |
| *D* | 5.88 | 1 | 5.88 | 25.59 | 0.0001 | ** |
| *AB* | 7.63 | 1 | 7.63 | 33.20 | < 0.0001 | ** |
| *AC* | 12.63 | 1 | 12.63 | 54.95 | < 0.0001 | ** |
| *AD* | 11.06 | 1 | 11.06 | 48.09 | < 0.0001 | ** |
| *BC* | 1.19 | 1 | 1.19 | 5.17 | 0.0381 | * |
| *BD* | 40.78 | 1 | 40.78 | 177.38 | < 0.0001 | ** |
| *CD* | 0.3790 | 1 | 0.3790 | 1.65 | 0.2186 |  |
| *A*^2^ | 0.2280 | 1 | 0.2280 | 0.9916 | 0.3351 |  |
| *B*^2^ | 4.47 | 1 | 4.47 | 19.42 | 0.0005 | ** |
| *C*^2^ | 1.11 | 1 | 1.11 | 4.83 | 0.0441 | * |
| *D*^2^ | 4.42 | 1 | 4.42 | 19.22 | 0.0005 | ** |
| Residual | 3.45 | 15 | 0.2299 |  |  |  |
| Loss of quasi item | 2.94 | 10 | 0.2938 | 2.88 | 0.1275 | not significant |
| Pure error | 0.5106 | 5 | 0.1021 |  |  |  |
| Total | 155.57 | 29 |  |  |  |  |

Note: "**" means very significant difference (P<0.01); "*" means significant difference (P<0.05)

**Supplementary Table 7.** Validation experimentation with single factor and optimal conditions of response surface

|  | Extraction temperature | material-liquid ratio | Ultrasonic time | Extraction time |
| --- | --- | --- | --- | --- |
| single factor | 70 | 1:35 | 25 | 60 |
| response surface | 75 | 1:35 | 25 | 74 |

**Supplementary** Table 8. Program and experimental results of RSM

| test number | A | B | C | D | A410/A490 | Extraction ratio /% |
| --- | --- | --- | --- | --- | --- | --- |
| 1 | 0.000 | 0.000 | 0.000 | 0.000 | 1.983 | 58.280 |
| 2 | 0.000 | 0.000 | 0.000 | 0.000 | 2.003 | 59.153 |
| 3 | 2.000 | 0.000 | 0.000 | 0.000 | 2.002 | 59.113 |
| 4 | 1.000 | -1.000 | 1.000 | 1.000 | 2.281 | 76.976 |
| 5 | 0.000 | 0.000 | 0.000 | 0.000 | 1.933 | 54.046 |
| 6 | -1.000 | 1.000 | -1.000 | -1.000 | 1.860 | 52.298 |
| 7 | 1.000 | 1.000 | -1.000 | 1.000 | 2.179 | 53.495 |
| 8 | 0.000 | 0.000 | 0.000 | 0.000 | 2.040 | 54.395 |
| 9 | -1.000 | -1.000 | -1.000 | 1.000 | 2.092 | 61.465 |
| 10 | 1.000 | 1.000 | 1.000 | 1.000 | 2.287 | 72.903 |
| 11 | 0.000 | 0.000 | 2.000 | 0.000 | 2.181 | 73.360 |
| 12 | 0.000 | -2.000 | 0.000 | 0.000 | 1.941 | 76.109 |
| 13 | 1.000 | -1.000 | 1.000 | -1.000 | 1.847 | 63.011 |
| 14 | 0.000 | 0.000 | 0.000 | 0.000 | 2.007 | 46.089 |
| 15 | 0.000 | 0.000 | -2.000 | 0.000 | 1.972 | 32.016 |
| 16 | -1.000 | -1.000 | 1.000 | -1.000 | 1.824 | 61.371 |
| 17 | 1.000 | -1.000 | -1.000 | -1.000 | 1.826 | 58.810 |
| 18 | -1.000 | 1.000 | -1.000 | 1.000 | 2.095 | 46.843 |
| 19 | 1.000 | 1.000 | 1.000 | -1.000 | 1.937 | 50.105 |
| 20 | -1.000 | -1.000 | -1.000 | -1.000 | 1.821 | 53.542 |
| 21 | -1.000 | 1.000 | 1.000 | 1.000 | 2.328 | 71.791 |
| 22 | 1.000 | 1.000 | -1.000 | -1.000 | 1.949 | 44.575 |
| 23 | 0.000 | 0.000 | 0.000 | -2.000 | 1.796 | 70.993 |
| 24 | 0.000 | 0.000 | 0.000 | 2.000 | 2.537 | 64.612 |
| 25 | -1.000 | 1.000 | 1.000 | -1.000 | 1.949 | 45.111 |
| 26 | -2.000 | 0.000 | 0.000 | 0.000 | 1.984 | 46.288 |
| 27 | 0.000 | 0.000 | 0.000 | 0.000 | 2.002 | 47.307 |
| 28 | 1.000 | -1.000 | -1.000 | 1.000 | 2.216 | 61.660 |
| 29 | -1.000 | -1.000 | 1.000 | 1.000 | 2.390 | 76.641 |
| 30 | 0.000 | 2.000 | 0.000 | 0.000 | 2.019 | 41.033 |

**Supplementary** Table 9. A410/A490 response surface analysis scheme and experiment results

| Soruce of variation | Quadratic sum | Degree of freedom | Mean square | F value | Pro>F | Significance |
| --- | --- | --- | --- | --- | --- | --- |
| Model | 0.9674 | 14 | 0.0691 | 68.6 | < 0.0001 | Significant |
| A | 0.0016 | 1 | 0.0016 | 1.63 | 0.2216 |  |
| B | 0.0082 | 1 | 0.0082 | 8.17 | 0.012 | * |
| C | 0.0624 | 1 | 0.0624 | 61.95 | < 0.0001 | ** |
| D | 0.7841 | 1 | 0.7841 | 778.4 | < 0.0001 | ** |
| AB | 0.0004 | 1 | 0.0004 | 0.3627 | 0.556 |  |
| AC | 0.0122 | 1 | 0.0122 | 12.08 | 0.0034 | ** |
| AD | 0.0002 | 1 | 0.0002 | 0.1533 | 0.7009 |  |
| BC | 0.0001 | 1 | 0.0001 | 0.0526 | 0.8217 |  |
| BD | 0.0136 | 1 | 0.0136 | 13.5 | 0.0023 | ** |
| CD | 0.0227 | 1 | 0.0227 | 22.55 | 0.0003 | ** |
| A2 | 1.74x10-6 | 1 | 1.74 x10-6 | 0.0017 | 0.9674 |  |
| B2 | 0.0003 | 1 | 0.0003 | 0.3295 | 0.5745 |  |
| C2 | 0.0117 | 1 | 0.0117 | 11.62 | 0.0039 | ** |
| D2 | 0.051 | 1 | 0.051 | 50.63 | < 0.0001 | ** |
| Residual | 0.0151 | 15 | 0.001 |  |  |  |
| Loss of quasi item | 0.0088 | 10 | 0.0009 | 0.7015 | 0.7045 | not significant |
| Pure error | 0.0063 | 5 | 0.0013 |  |  |  |
| Total | 0.9825 | 29 |  |  |  |  |

**Supplementary** Table 10. DPPH clearance rate response surface analysis scheme and experiment results

| Soruce of variation | Quadratic sum | Degree of freedom | Mean square | F value | Pro>F | Significance |
| --- | --- | --- | --- | --- | --- | --- |
| Model | 3131.98 | 14 | 223.71 | 4.1 | 0.0051 | Significant |
| A | 60.56 | 1 | 60.56 | 1.11 | 0.3089 |  |
| B | 894.35 | 1 | 894.35 | 16.38 | 0.0011 | ** |
| C | 1174.69 | 1 | 1174.69 | 21.52 | 0.0003 | ** |
| D | 267.91 | 1 | 267.91 | 4.91 | 0.0426 | * |
| AB | 0.3612 | 1 | 0.3612 | 0.0066 | 0.9362 |  |
| AC | 0.8508 | 1 | 0.8508 | 0.0156 | 0.9023 |  |
| AD | 1.06 | 1 | 1.06 | 0.0194 | 0.8911 |  |
| BC | 0.002 | 1 | 0.002 | 0 | 0.9953 |  |
| BD | 10.46 | 1 | 10.46 | 0.1916 | 0.6679 |  |
| CD | 259.83 | 1 | 259.83 | 4.76 | 0.0455 | * |
| A2 | 0.3785 | 1 | 0.3785 | 0.0069 | 0.9347 |  |
| B2 | 68.92 | 1 | 68.92 | 1.26 | 0.2789 |  |
| C2 | 0.3593 | 1 | 0.3593 | 0.0066 | 0.9364 | ** |
| D2 | 415.71 | 1 | 415.71 | 7.61 | 0.0146 | ** |
| Residual | 818.91 | 15 | 54.59 |  |  |  |
| Loss of quasi item | 670.23 | 10 | 67.02 | 2.25 | 0.1913 | not significant |
| Pure error | 148.68 | 5 | 29.74 |  |  |  |
| Total | 3950.9 | 29 |  |  |  |  |

**Supplementary** Table 11 .Single factor and response surface optimal condition validation test

|  | Ultrasonic time  /min | Polysaccharide concentration  /mg·mL^-1^ | Sodium selenite concentration  / mg·mL^-1^ | Selenide temperature  /℃ |
| --- | --- | --- | --- | --- |
| Single factor  (clearance rate) | 10 | 10 | 12 | 30 |
| Single factor  (stability) | 15 | 14 | 12 | 80 |
| Response surface | 10 | 10 | 12 | 50 |

**Supplementary Figure1.** Elution curve of chestnut polysaccharide on DEAE-52 cellulose ion exchange column

**Supplementary Figure 2**. Elution curve of chestnut polysaccharide on Sephadex G-100 glucan gel column

**（B）**

**（A）**

**Supplementary** **Figure 3.** Molecular weight determination of CP (A) and CP(B)

**Supplementary Figure 4.** Chromatograms of standard monosaccharides

**（A）**

**（B）**

**Supplementary Figure 5.** Monosaccharide composition of CP (A) and CP-1A (B)

**Supplementary Figure 6.** UV spectra of CP, CP-1 and CP-1A
